# Healthcare workers knowledge of cholera multi-stranded interventions and its determining factors in North-East Nigeria: planning and policy implications

**DOI:** 10.1186/s12960-023-00796-7

**Published:** 2023-02-01

**Authors:** Kelly Elimian, Carina King, Ozius Dewa, Emmanuel Pembi, Benjamin Gandi, Sebastian Yennan, Puja Myles, Catherine Pritchard, Birger Carl Forsberg, Tobias Alfvén

**Affiliations:** 1 Department of Global Public Health, Karolinska Institutet, Stockholm, Sweden; 2 Exhale Health Foundation, Abuja, Nigeria; 3 School of Health Systems and Public Health, University of Pretoria, Pretoria, South Africa; 4 Adamawa State Ministry of Health, Yola, Adamawa State Nigeria; 5 Bauchi State Ministry of Health, Yola, Bauchi State Nigeria; 6 Nigeria Centre for Disease Control, Abuja, Nigeria; 7 Clinical Practice Research Datalink, Medicines and Healthcare Products Regulatory Agency, London, UK; 8 Public Health Division, Nottinghamshire County Council, Nottingham, UK; 9 Sachs’ Children and Youth Hospital, Stockholm, Sweden

**Keywords:** Cholera, Knowledge, Healthcare workers, Interventions, Conflict, Nigeria

## Abstract

**Background:**

Healthcare workers’ (HCWs) knowledge of multi-stranded cholera interventions (including case management, water, sanitation, and hygiene (WASH), surveillance/laboratory methods, coordination, and vaccination) is crucial to the implementation of these interventions in healthcare facilities, especially in conflict-affected settings where cholera burden is particularly high. We aimed to assess Nigerian HCWs’ knowledge of cholera interventions and identify the associated factors.

**Methods:**

We conducted a cross-sectional study using a structured interviewer-administered questionnaire with HCWs from 120 healthcare facilities in Adamawa and Bauchi States, North-East Nigeria. A knowledge score was created by assigning a point for each correct response. HCWs’ knowledge of cholera interventions, calculated as a score, was recoded for ease of interpretation as follows: 0–50 (low); 51–70 (moderate); ≥ 71 (high). Additionally, we defined the inadequacy of HCWs’ knowledge of cholera interventions based on a policy-relevant threshold of equal or lesser than 75 scores for an intervention. Multivariable logistic regression was used to identify the factors associated with the adequacy of knowledge score.

**Results:**

Overall, 490 HCWs participated in the study (254 in Adamawa and 236 in Bauchi), with a mean age of 35.5 years. HCWs’ knowledge score was high for surveillance/laboratory methods, moderate for case management, WASH, and vaccination, and low for coordination. HCWs’ knowledge of coordination improved with higher cadre, working in urban- or peri-urban-based healthcare facilities, and secondary education; cholera case management and vaccination knowledge improved with post-secondary education, working in Bauchi State and urban areas, previous training in cholera case management and response to a cholera outbreak—working in peri-urban areas had a negative effect. HCWs’ knowledge of surveillance/laboratory methods improved with a higher cadre, 1-year duration in current position, secondary or post-secondary education, previous training in cholera case management and response to a cholera outbreak. However, HCWs’ current position had both positive and negative impacts on their WASH knowledge.

**Conclusions:**

HCWs in both study locations recorded a considerable knowledge of multi-stranded cholera interventions. While HCWs’ demographic characteristics appeared irrelevant in determining their knowledge of cholera interventions, geographic location and experiences from the current position, training and involvement in cholera outbreak response played a significant role.

**Supplementary Information:**

The online version contains supplementary material available at 10.1186/s12960-023-00796-7.

## Background

Nigeria has experienced several cholera outbreaks in recent decades, recording over 40,000 cholera cases and 1700 deaths in 2010 [1] and over 50,000 cases and 800 deaths in 2018 [2]. Between October 2020 and October 2021, amidst the COVID-19 pandemic, the country experienced an unprecedented cholera outbreak which spanned across 33 of its 37 states (including the Federal Capital Territory), recording over 93,000 cholera cases and 3000 deaths (case fatality rate: 4%) [3]. Like the previous cholera outbreaks, the northern region of Nigeria, particularly the North-East region, disproportionately accounted for a higher burden from the outbreak. Consistent with existing literature [4, 5], the substantial burden of the cholera outbreak in the North-East was partly attributed to the Boko Haram insurgency [3], which has exacerbated the already poor water, sanitation, and hygiene (WASH) services, living conditions and population congestion in the region [6]. Insurgency in the region is also believed to have weakened the Integrated Disease Surveillance and Response (IDSR) system for infectious diseases, especially regarding surveillance data collection and transmission at the community level, leading to an underestimation of disease burden [7].

The World Health Organization (WHO) Global Task Force on Cholera Control (GTFCC) coordinates a concerted response to the increasing global cholera burden. This response is hinged on effectively implementing multi-stranded cholera interventions in order to actualise the GTFCC’s strategic roadmap aims for a 90% reduction in cholera deaths and disease elimination in at least half of cholera endemic countries by 2030 [8]. These multi-stranded cholera interventions include WASH, surveillance/laboratory methods, oral cholera vaccination (OCV), case management, leadership and coordination, and community engagement. An assessment of the HCWs’ knowledge is crucial as they have a critical role to play in the successful implementation of these interventions. The report of nosocomial cholera infections among HCWs during an outbreak in northern Nigeria [9] underscores the significance of their knowledge of cholera interventions for infection prevention and control. HCWs’ knowledge of cholera interventions can be beneficial for establishing a transparent system of leadership and coordination for cholera outbreak response, ensuring efficiencies in implementing process, and strengthening human resource and health systems functions for anticipatory preparedness [10]. For example, a rapid assessment of cholera knowledge deficiencies among HCWs was strategic in informing the Dominican Republic’s preparedness (immediate training and development of educational materials) for the 2010 cholera epidemic occurring in neighbouring Haiti [11].

The Nigerian Government and its development partners have made reasonable investments in training HCWs on multi-stranded cholera interventions, especially case management and surveillance in anticipation of a cholera outbreak. However, there seems to be a lack of formal evaluation of the impacts of such investments, which could be measured by HCWs’ knowledge of the multi-stranded cholera interventions and their associated factors in the context of health systems vulnerable to armed conflict. Moreover, there is a dearth of evidence on HCWs’ knowledge of cholera multi-stranded interventions and its determining factors, with the few studies available being community-based [12–16]. A cross-sectional survey of 286 HCWs in health facilities in Oyo State, South-West Nigeria, found inadequate knowledge of general cholera prevention and management procedures, with 83.6% not knowing the cholera alert threshold [17].

Knowledge assessment is one of the most challenging tasks due to the complexity and invisible nature of knowledge [18]. The literature offers frameworks that make an effort to assess knowledge at different levels and from different perspectives, such as human resources management [19] and organisational intellectual capital [20]. However, an examination of existing frameworks indicates that they have an organisational perspective on knowledge and do not handle knowledge assessment on the individual level [21]. Overall, the knowledge of healthcare professionals is inextricably related to their unique capacities to produce, disseminate, and use knowledge, thus enhancing the capacity of the healthcare system to withstand and react to disasters as well as implement relevant interventions sustainably. We aimed to fill the identified conceptual and practice gaps by assessing HCWs’ individual knowledge of cholera multi-stranded interventions and their determining factors in a cholera endemic conflict-ridden area of Nigeria. Findings from the study will be crucial to contextualising interventions to improve HCWs’ knowledge thus enhancing the implementation of health system resilience strengthening multi-stranded cholera interventions in the area.

## Methods

### Study design

We conducted a cross-sectional survey using an interviewer-administered structured questionnaire. This study is part of a larger study focused on strengthening Nigeria’s capacity for response to cholera outbreaks in North-East Nigeria [22].

### Study setting

Nigeria consists of 36 States and the Federal Capital Territory (Abuja). However, this study was conducted in Adamawa and Bauchi States in North-East Nigeria because of cholera endemicity [2] and fragility with direct implications for cholera transmission. Bauchi State has about 7.5 million people across 20 Local Government Areas (LGAs), while Adamawa State has an estimated population of 4.7 million across 21 LGAs [23]. In addition to serving as a host community to several internally displaced persons (IDPs) from Boko Haram’s primary hubs (Borno and Yobe States in the North-East), few Adamawa State LGAs have been directly attacked by Boko Haram in the past. Overall, the North-East region has the lowest access to potable water supply and sanitation services at 2% as compared with the South-West with the highest access at 31% [6].

### Sample size estimation, study population, and sampling

Nigeria’s healthcare sector strategic plans aim to ensure that HCWs that are qualified, skilled, and distributed equitably are available for quality healthcare service provision at all levels of care (primary, secondary, and tertiary) [24]. There were 760 healthcare facilities (345 in Adamawa and 415 in Bauchi) considered functional and safe during the study period. Of these, 120 healthcare facilities were purposefully sampled based on managers formally expressing willingness to participate. We deliberately tried to sample contrasting settings (rural, peri-urban, and urban areas), given the effect on cholera endemicity [2]. Within each sampled facility, we systematically sampled 3–4 consenting HCWs per healthcare facility for the study. The process involved selecting every third HCW that walks into the reception of the healthcare facility on the day of data collection. The overreaching study, for which this was a component, did not formally estimate sample size due to pragmatic constraints dictated by the study setting (e.g., staff safety, travel and time).

### Data collection and tools

Before data collection, we conducted a 2-day training of 7 research assistants in each state. The research assistants were disease notification and surveillance officers and contact tracers for COVID-19 response in the states. The training covered the study-specific objectives and methodology, ethics, data quality, data collection using an Open Data Kit Application [25], and infection prevention and control (IPC) measures given the COVID-19 pandemic. The data collection protocol was updated following piloting with HCWs at a healthcare facility in Abuja to assess the questions' completeness, clarity, and accuracy.

The data collection tool was developed by adapting existing tools for assessing service provision in healthcare facilities, including the “WHO core questions and indicators for monitoring WASH in HCFs” [26], the “WHO guide for assessing cholera outbreak response and improving preparedness” [27], and the “WHO service availability and readiness assessment tool” [28]. The data collection tool was given to two local infectious disease experts for review before deployment; it consisted of two sections. The first section covered sociodemographic characteristics of the HCWs and a description of the healthcare facility. The second section covered HCWs’ knowledge of the various cholera interventions. See details of the study questionnaire in Additional file 1. Following administrative approval to undertake data collection, a research assistant met with a selected HCW to administer the questionnaire on an agreed time. Data collection was conducted from 8 to 30 March 2021.

### Definition of outcome variables and covariates

The first outcome variable is HCWs’ knowledge of cholera interventions, which was calculated as a score (continuous variable). However, it was recoded for ease of interpretation: 0–50 (low); 51–70 (moderate); ≥ 71 (high). The second outcome variable is the adequacy of HCWs’ knowledge of cholera interventions, which was also recoded from continuous to a binary variable using a policy-relevant threshold. While a policy-relevant threshold of greater than 80 scores has been reported for COVID-19 [29–31], the Nigeria national cholera technical working group advised using a slightly lower cut-off value of 75. Therefore, adequate knowledge of a cholera intervention was defined as a score greater than 75 (coded 0), and inadequate knowledge as a score equal to or lesser than 75 for the intervention (coded 1). We pre-selected variables conceptually considered associated with the second outcome variable based on previous research [32, 33], biological plausibility and data availability (see Table 1 for definitions).

**Table 1:** Definition of covariates

Variable	Definition variable/type
Age, in year	Based on self-reports by the study participants and retained as a continuous variable due to the *p*-value (*p* > 0.05) obtained from the Likelihood Ratio Test (LRT)
Sex	Binary variable: female vs male
Health facility type	Categorical variable: primary; secondary; and tertiary
Setting	Categorical variable: rural, urban, and peri-urban. An LGA was classified as urban if at least two of the following criteria were met: State capital; estimated population size of ≥ 20,000; > 75% of its population is engaged in non-agricultural occupations; availability of infrastructure (e.g., hospital, schools), functional transportation system, and a broad array of economic, social and recreational activities [34]. However, an LGA without distinctive urban or rural features was classified as peri-urban
Highest level of education completed	Categorical variable: primary; secondary; post-secondary/tertiary; and other
Current position	Categorical variable, grouped as follows: Community Health Extension Worker (CHEW)/attendant/cleaner/casual staff (1); junior staff (e.g., disease focal person/dispenser; (2); nurse/laboratorian/data scientist (3); and clinician/pharmacist senior administrative or senior staff (4)
Previous training in cholera management	Binary variable (No vs Yes). Cholera case management refers to managing cholera patients and other related activities, including surveillance and infection prevention and control
Participation in a previous cholera outbreak response	Binary variable: No vs Yes. We did not specify the timeline; instead, we focused on anytime period

### Data management

We adopted a scoring system previously used in Malawi [35]. A score of '1' was assigned to the 'correct' response and '0' for the ‘incorrect’ response. The individual scores were then summed up based on all the responses within an intervention to derive the total score for each cholera intervention. For multiple-response questions with cholera case management, a point was given for each correct response, giving a total score of 42, based on 14 questions. Scores were then converted to percentages. This process was repeated for WASH with a total score of 14, surveillance with 20, coordination with 8, and OCV with 4. The calculated mean scores (normally distributed) of each cholera intervention were then used for radar plotting to depict HCWs' knowledge of each cholera intervention pictorially. The radar plot was categorised as follows: 0, 10, 20, 30, 40, 50, 60, 70, 80, 90, and 100; the closer the edge of a cholera intervention is to 100, the better the knowledge of that intervention, and vice-versa.

### Statistical analysis

Baseline characteristics of HCWs and health facilities were described by study locations (Adamawa and Bauchi States) using mean and standard deviation (SD) for normally distributed continuous variables and frequency and percentages (%) for categorical/binary variables. HCWs’ knowledge of multi-stranded cholera interventions was described using mean and SD because of its normal distribution. HCWs’ sociodemographic and health facility characteristics in relation to knowledge of cholera interventions were described using Chi-squared tests, and a *p*-value of < 0.05 was considered statistically significant. To identify covariates associated with inadequate knowledge of each cholera intervention, unadjusted logistic regression analyses were performed, and the findings were presented as unadjusted odds ratios (ORs) with 95% confidence intervals (CIs). The likelihood ratio test (LRT) p-values were presented for categorical variables, while Wald's *p*-value was used for binary/continuous variables. These analyses were followed by multivariable analyses (separately for each cholera intervention) using a stepwise multiple logistic regression to identify the significant determinants of inadequate knowledge of cholera interventions among HCWs. Findings from the multivariable model were presented as adjusted ORs with 95% CIs. All analyses were performed using Stata 16 (Stata Corp. LP, College Station, TX, United States of America). This paper was written following the Strengthening the Reporting of Observational Studies in Epidemiology (STROBE) checklist for cross-sectional studies (Additional file 2).

## Results

### Baseline characteristics of the study participants

Overall, 490 HCWs (254 in Adamawa State and 236 in Bauchi State) participated in the study (Table 2). The mean age of HCWs was 35.5 years. Most of the HCWs in both states were working in primary healthcare centres and government-owned healthcare facilities. Most HCWs in Adamawa State worked in urban areas (78.7%, 200/254), whereas the majority in Bauchi State worked in rural areas (76.3%, 180/236).

**Table 2:** Baseline characteristics of HCWs assessed for knowledge regarding cholera interventions

Variable	Adamawa State [*n* = 254]	Bauchi State [*n* = 236]	Overall [*n* = 490]
Local Government Area	3	8	11
Mean (SD) age, year	33.67 (8.1)	37.38 (7.3)	35.46 (8.0)
*Sex*			
Female	142 (55.9)	86 (36.4)	228 (46.5)
Male	112 (44.1)	150 (63.6)	262 (53.5)
*Health facility type*			
Primary	208 (81.9)	208 (88.1)	416 (84.9)
Secondary	36 (14.2)	28 (11.9)	64 (13.1)
Tertiary	10 (3.9)	-	10 (2.0)
*Setting*			
Rural	37 (14.6)	180 (76.3)	217 (44.3)
Urban	200 (78.7)	25 (10.6)	225 (45.9)
Peri-urban	17 (6.7)	31 (13.1)	48 (9.8)
*Highest level of education completed*			
Primary	7 (2.8)	70 (29.7)	77 (15.7)
Secondary	31 (12.2)	19 (8.1)	50 (10.2)
Post-secondary/tertiary	214 (84.3)	144 (61.0)	358 (73.1)
Other	2 (0.8)	3 (1.3)	5 (1.0)
*Current position (cadre)$*			
1	100 (39.4)	88 (37.3)	188 (38.4)
2	69 (27.2)	32 (13.6)	101 (20.6)
3	49 (19.3)	25 (10.6)	74 (15.1)
4	36 (14.2)	91 (38.6)	127 (25.9)
Mean (SD) year in current position	4.21 (4.0)	16.23 (11.9)	10.00 (10.6)
*Previous training in cholera management*			
No	194 (76.4)	178 (75.4)	372 (75.9)
Yes	60 (23.6)	58 (24.6)	118 (24.1)
*Participation in a previous cholera outbreak*			
No	102 (40.2)	113 (47.9)	215 (43.9)
Yes	152 (59.8)	123 (52.1)	275 (56.1)

SD: standard deviation, NGO: non-governmental organisation, CHEW: Community Health Extension Worker

$Current position: 1 = CHEW/attendant/cleaner/casual staff; 2 = junior (disease focal person) staff; 3 = nurse/laboratorian/data scientist; 4 = senior: clinician/pharmacist/administrative staff

**Fig. 1:**
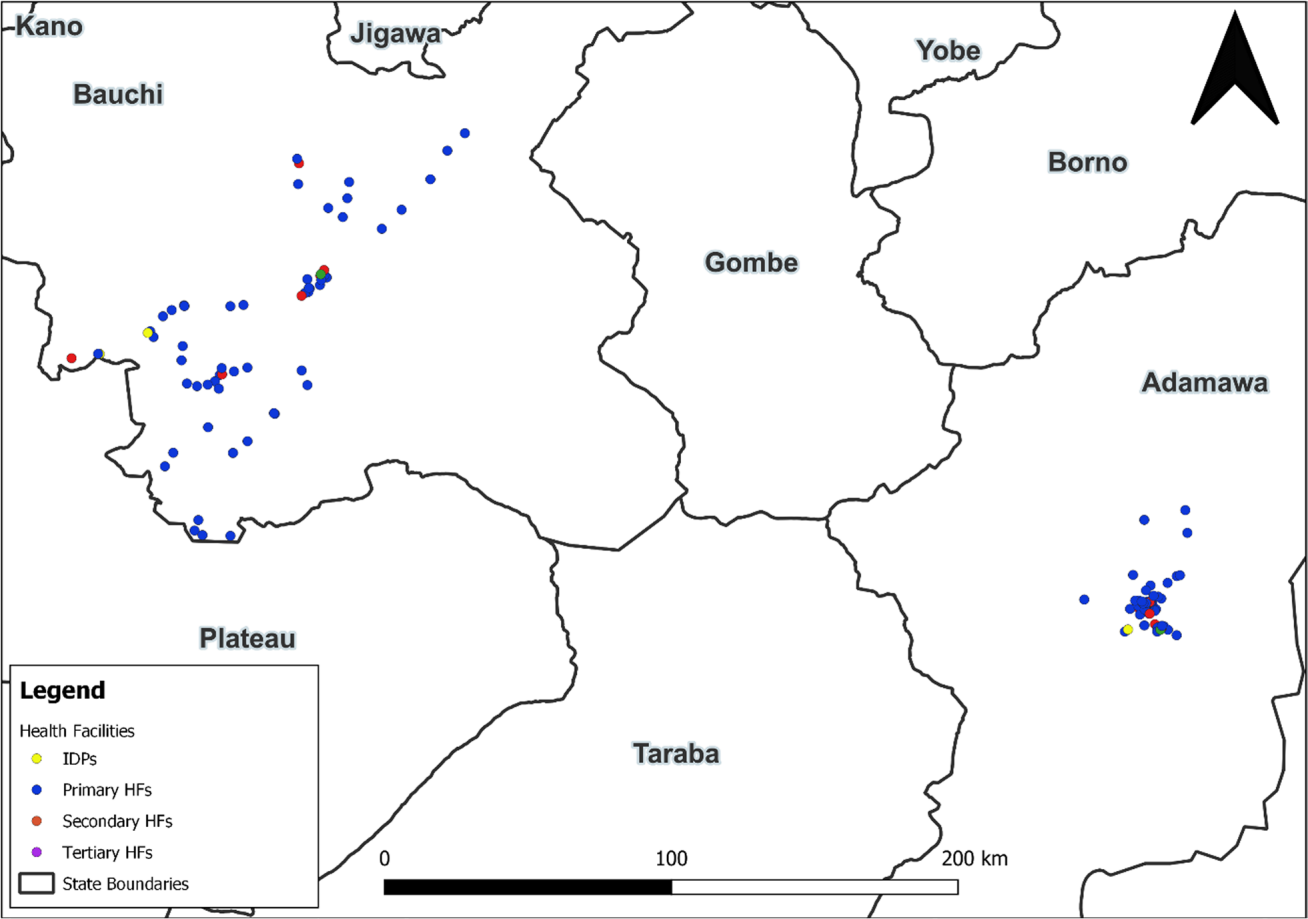
Spatial distribution of healthcare facilities in Adamawa and Bauchi States

Figure 1 is a Nigerian map showing the distribution of the study healthcare facilities in Adamawa and Bauchi States.

### HCWs’ knowledge of cholera interventions (first outcome variable)

**Fig. 2:**
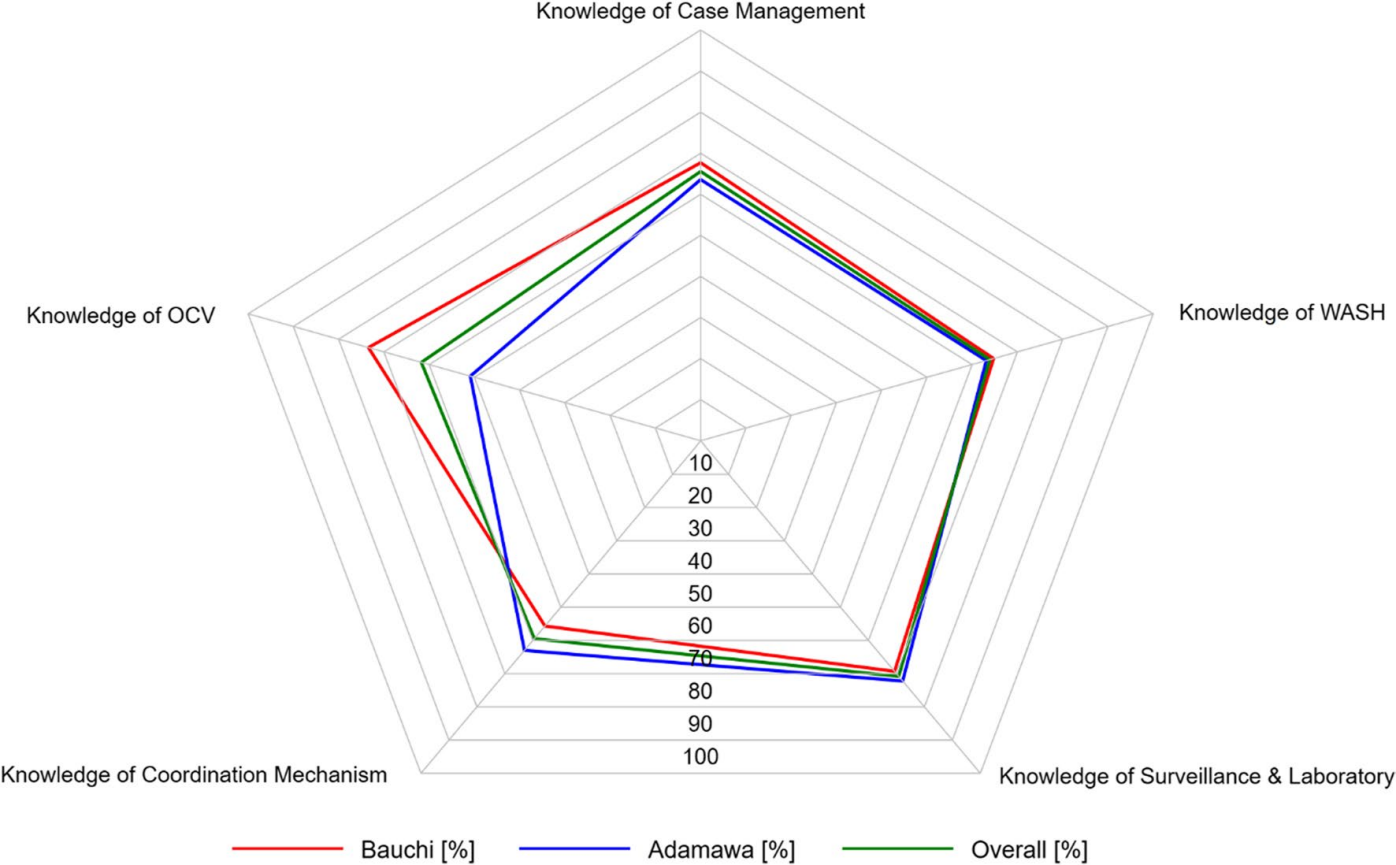
A radar plot showing healthcare workers’ knowledge of cholera interventions in Adamawa and Bauchi States, Nigeria

Overall, the highest and lowest mean knowledge scores recorded by HCWs were for surveillance and laboratory (70.8) and coordination (59.5), respectively; however, they recorded moderate mean knowledge scores for case management (65.6), WASH (63.9), and vaccination (61.7) (Table 3). Similar to the overall pattern, the highest mean knowledge score by HCWs in Adamawa State was for surveillance and laboratory at 72.2. However, the highest mean knowledge score by HCWs in Bauchi State was for vaccination at 73.4. HCWs in Bauchi State were more knowledgeable in case management, vaccination and WASH than their counterparts in Adamawa State. A radar plot depicting HCWs’ knowledge of multi-stranded cholera interventions mirrors the mean knowledge scores (Fig. 2).

**Table 3:** Healthcare workers’ mean knowledge scores for cholera multi-stranded interventions in Adamawa and Bauchi States

Cholera intervention	Adamawa State [*n* = 254]		Bauchi State [*n* = 236]		Overall [*N* = 490]	
	Mean (SD)	Range score [lowest/highest]	Mean (SD)	Range score [lowest/highest]	Mean (SD)	Range score [lowest/highest]
Case management	63.6 (16.4)	21.4–92.9	67.7 (12.3)	31.0- 85.7	65.6 (14.7)	21.4- 92.9
Water, sanitation, and hygiene	63.1 (11.3)	21.4–100.0	64.7 (12.1)	21.4–85.7	63.9 (11.7)	21.4–100.0
Surveillance and laboratory	**72.2 (17.1)**	25.0–100.0	69.3 (17.8)	30.0–95.0	**70.8 (17.4)**	25.0–100.0
Coordination	63.0 (23.6)	0.0–100.0	55.7 (25.0)	0.0–100.0	59.5 (24.5)	0.0–100.0
Oral cholera vaccine	50.9 (24.1)	0.0–100.0	**73.4 (23.5)**	0.0–100.0	61.7 (26.3)	0.0–100.0

The highest values are in bold

### Factors associated with HCWs’ knowledge of multi-stranded cholera interventions

The association between HCWs’ characteristics and adequacy of knowledge of cholera interventions is presented in Additional file 3; the unadjusted ORs for the association between covariates and HCWs’ knowledge of cholera interventions are presented in Additional file 4. Table 4 presents the adjusted ORs for covariates associated with HCWs’ knowledge of cholera interventions.

**Table 4:** Factors associated with inadequate knowledge of cholera interventions

Variable	Case management	WASH	Surveillance and laboratory	Coordination	Oral cholera vaccine
	Adjusted OR (95% CI)				
*State*					
Adamawa	–	–	–	–	1.00
Bauchi					**0.03 (0.01–0.07)** ‡
Age, year	–	–	0.99 (0.96–1.03) NS	–	0.99 (0.96–1.04) NS
*Sex*					
Female	1.00	–	–	1.00	–
Male	1.51 (0.93–2.47) NS			1.22 (0.76–1.97) NS	
*Health facility type*					
Primary	–	–	1.00	1.00	–
Secondary			0.82 (0.43–1.58)	**0.45 (0.25–0.87)**	
Tertiary			1.36 (0.31–6.02) NS	0.46 (0.11–1.84) †	
*Setting*					
Rural	1.00	–	1.00	1.00	1.00
Urban	1.49 (0.89–2.50)		0.75 (0.43–1.29)	**0.51 (0.31–0.86)**	**0.20 (0.07–0.53)**
Peri-urban	**3.59 (1.25–10.35)** †		1.04 (0.47–2.31) NS	**0.39 (0.18–0.83)** †	**3.54 (1.22–10.25)** †
*Highest level of education*					
Primary	–	–	1.00	1.00	1.00
Secondary			**0.07 (0.02–0.28)**	0.58 (0.15–1.97)	1.08 (0.16–7.36)
Post-secondary/tertiary			**0.06 (0.02–0.23)**	0.46 (0.14–1.46)	**0.12 (0.04–0.36)**
Other			0.31 (0.02–3.80) †	3.63 (0.20–64.72) NS	0.10 (0.01–1.04) †
*Current position (cadre)*^$^					
1	1.00	1.00	1.00	1.00	1.00
2	**0.11 (0.06–0.22)**	**4.55 (1.72–11.99)**	**0.08 (0.04–0.15)**	**0.07 (0.04–0.14)**	1.45 (0.58–3.64)
3	0.93 (0.39–2.22)	**2.68 (1.08–6.67)**	**0.34 (0.18–0.68)**	0.71 (0.33–1.52)	2.71 (0.95–7.72)
4	**0.44 (0.23–0.87)** †	**3.11 (1.44–6.70)** †	**0.20 (0.11–0.38)** †	**0.22 (0.12–0.41)** †	1.82 (0.71–4.70) NS
Duration in current position, year	–	0.98 (0.96–1.00) NS	**0.95 (0.92–0.98)** †	0.98 (0.95–1.02) NS	–
*Previous training in cholera management*					
No	1.00	–	1.00	1.00	1.00
Yes	**0.42 (0.25–0.71) †**		**0.40 (0.23–0.69)** †	0.61 (0.35–1.05) NS	**0.37 (0.20–0.70)** †
*Participation in a previous cholera outbreak*	1.00	–	1.00	1.00	1.00
No	**0.39 (0.22–0.70)** †		**0.58 (0.35–0.94)** †	0.60 (0.34–1.05) NS	**0.49 (0.24–0.99)** †
Yes					

Wald’s* p*-values are presented for binary and continuous variables; LRT p-values are presented for categorical variables

Statistically significant results are in bold

† = *p*-value < 0.05; ‡ = *p*-value < 0.001; ns = *p*-value not statistically significant (i.e.  ≥ 0.05)

^$^Current position: 1 = CHEW/attendant/cleaner/casual staff; 2 = junior (disease focal person/dispenser) staff; 3 = nurse/laboratorian/data scientist; 4 = senior: clinician/administrative staff/chief nurse

#### Case management

HCWs’ knowledge of cholera case management remained significantly associated with setting, current position, previous training in cholera case management and participation in a cholera outbreak. Specifically, HCWs’ odds of inadequate knowledge of cholera case management was almost fourfold higher with working in peri-urban areas than in rural areas (referent group). In contrast, their odds of inadequate knowledge of cholera case management was 89% and 66% lower if working as a junior (e.g., disease focal person/dispenser) and senior (e.g., clinician, administrative/chief nurse) staff, respectively, in comparison to working as CHEW/attendant. Additionally, HCWs’ odds of inadequate knowledge of cholera case management significantly lowered with previous training in cholera case management (adjusted OR 0.42; 95%: 0.25–0.71) and participation in a cholera outbreak response (adjusted OR 0.39; 95% CI: 0.22–0.70) when compared with colleagues without such training and experience.

#### WASH

Only HCWs’ current position was significantly associated with their knowledge of WASH services within the context of cholera, with high-cadre positions recording increased odds of inadequate knowledge. Specifically, HCWs’ odds of inadequate knowledge of WASH was about sixfold higher if working as a junior and threefold higher each if working as nurse/laboratorian and as senior (e.g., clinician, administrative staff) staff than working as CHEW/attendant.

### Surveillance and laboratory

HCWs’ odds of inadequate knowledge of cholera surveillance and laboratory diagnostics significantly decreased as low as 93% with secondary education and 94% with post-secondary education compared to primary education. Working as a junior staff, nurse/laboratorian/data scientist, and senior (clinician, administrative/chief nurse) staff significantly decreased the odds of inadequate knowledge of cholera surveillance and laboratory diagnostics by 92%, 66%, and 80%, respectively, when compared with working as CHEW/attendant staff. Additionally, previous training in cholera case management and previous participation in a cholera outbreak response significantly decreased HCWs’ odds of inadequate knowledge of cholera surveillance and laboratory diagnostics by 60% and 42%, respectively.

### Coordination

HCWs’ odds of inadequate knowledge of cholera outbreak coordination was 55% lower if working in a secondary hospital than in a primary healthcare facility. Compared to HCWs working in rural areas, those working in urban (adjusted OR 0.51; 95% CI: 0.31–0.86) and peri-urban (adjusted OR 0.39; 95% CI: 0.18–0.83) areas had a significantly lower odds ratio for inadequate knowledge of cholera outbreak coordination. Lastly, HCWs’ odds of inadequate knowledge of cholera outbreak response was 93% lower if working as junior staff and 82% lower if working as senior staff compared to CHEW/attendant.

#### Oral cholera vaccine

Unlike the other interventions, HCWs’ state of residence was significantly associated with HCWs’ knowledge of vaccination. Their odds of inadequate vaccination knowledge decreased by 97% if working in Bauchi State compared to Adamawa State. The setting of an HCW’s health facility had a contrasting effect on their knowledge of OCV as the odds of inadequate knowledge of vaccination was 80% lower among those working in urban areas but about fourfold higher among those working in peri-urban areas. Compared with HCWs with primary education, those with post-secondary/tertiary education had significantly lower odds of inadequate vaccination knowledge. Both previous training in cholera case management and response to a cholera outbreak significantly lowered the odds of inadequate vaccination knowledge.

## Discussion

This study contributes significantly to the available knowledge on the status of HCWs’ knowledge of cholera multi-stranded interventions and their associated factors, thus, addressing both conceptual and practice gaps observed in previous studies. We found that HCWs had high knowledge of cholera surveillance/laboratory methods, moderate knowledge of case management, WASH, and vaccination, and low knowledge of coordination. HCWs’ exposure to cholera outbreaks by virtue of health facility location and experiences from current position, as well as training and involvement in cholera outbreak response is significant in determining their knowledge of multi-stranded cholera interventions.

HCWs had a higher knowledge score for surveillance than the other cholera interventions, which could be partly explained by frequent engagement (active and inactive) in routine surveillance of endemic infectious diseases within the IDSR system in Nigeria. This is particularly likely as, unlike case management and vaccination, some of the study questions about surveillance (e.g., “surveillance allows healthcare workers to”) were generic. It is therefore possible for HCWs to infer the correct responses from engagement in other disease surveillance activities. HCWs recorded a moderate knowledge score for case management and vaccination, which is expected as the fundamental activities related to both interventions tend to be linked to cholera outbreak responses. It is therefore not surprising that HCWs in Bauchi State had higher knowledge scores and lower odds of inadequate knowledge of cholera vaccination than their Adamawa State counterparts. This is because Bauchi State has consistently accounted for higher absolute number of cholera cases than any other state in Nigeria in the latest cholera outbreaks in Nigeria [2, 3]. Expectedly, Bauchi State has witnessed more characterised by frequent vaccination campaigns than Adamawa State [36, 37]. Further, our findings indicate that HCWs’ knowledge of case management, coordination and vaccination could be influenced by the location of healthcare facility, with those working in urban areas more knowledgeable of coordination and vaccination than those in rural areas. This trend could be explained by the fact that cholera emergency operation centres are often situated and managed in the state capital (mostly urban) and HCWs in the state capital benefits more from training and mentorship measures organised by the Nigerian development partners (e.g., WHO, UNICEF, MSF, among others) than their colleagues in rural or peri-urban areas. However, the advantage in knowledge of cholera outbreak coordination that HCWs in peri-urban areas have over those in rural areas could be attributable to frequent encounter (and increased experience) with cholera outbreaks in Nigeria [2]. Unexpectedly, HCWs in peri-urban healthcare facilities were less knowledgeable about cholera case management and vaccination, underlining the need for increased investments of resources in such areas at high risk of a cholera outbreak [38, 39].

HCWs’ knowledge of cholera outbreak coordination at various levels of care is crucial to the implementation of all the multi-stranded cholera interventions, especially in tertiary hospitals where most of the cholera treatment centres are situated for central coordination and case management during a cholera outbreak [In Press]. In general, HCWs working at a higher level of care (secondary and tertiary hospitals) tend to benefit from more training and mentorship opportunities from diverse cholera stakeholders (government and development partners) than those working at the primary healthcare level. It is therefore surprising that HCWs working in tertiary hospitals did not record significantly lower odds of inadequate knowledge of cholera coordination compared to those working in primary healthcare centres (referral of suspected cholera patients at this level of care is recommended)—only those working in secondary hospitals had a significant advantage in this regard. However, the finding could be explained by the fact that only a handful of HCWs in tertiary hospitals are involved in cholera outbreak response, partly to promote infection prevention and control, which was not considered an inclusion criterion for this study.

HCWs with higher education, especially post-secondary or tertiary education, had significantly lower odds of inadequate knowledge (i.e. had knowledge advantage) of cholera surveillance and laboratory diagnostics and vaccination in the present study. This is consistent with a study conducted in Guinea Bissau, which found HCWs’ knowledge score for health interventions to improve significantly with higher education [40]. While a higher level of education is a prerequisite for attaining a higher professional cadre, the occupational cadre of HCWs in the present study showed a contrasting association with WASH intervention, with HCWs occupying more senior or specialised cadres recording higher odds of inadequate knowledge of WASH services than community-based staff (e.g., community health extension health worker and health attendant/cleaner). Although it is difficult to establish that knowledge of intervention would automatically translate to practice, adequate knowledge is an essential requirement for the translation process. Thus, the implementation of WASH services within a healthcare facility could be slow if the staff occupying strategic positions (and often leadership positions) are not conversant with the intervention. Conversely, HCWs occupying more specialised positions had significantly lower odds of inadequate knowledge of cholera case management, surveillance and laboratory, and coordination than community-based HCWs. These findings could be explained, in part, by the place of work and job specifications of HCWs, with those occupying specialised positions more likely to be working in tertiary or secondary hospitals (where cholera is typically managed) than community-based colleagues who predominantly work in primary healthcare facilities.

It is worth noting that clinicians and pharmacists did not demonstrate significantly lower odds of vaccination knowledge over community-based staff. However, this could be a function of the specialised scope of their roles rather than inadequate knowledge per se, which is likely in our study settings where Boko Haram insurgency has directly contributed to HCWs’ attrition [41]. Although not directly assessed in the present study, persistent armed conflict in the study setting can affect HCWs’ knowledge of cholera multi-stranded interventions. As argued by Burkle and colleagues, armed conflict could derail instructional opportunities in both content and preparation of HCWs to manage multiple challenges and threats and to perform triage management in a resource-poor medical setting [42]. Further, while the yearly stay in the current position of HCWs had no significant association with WASH and coordination, it decreased the odds of inadequate knowledge of cholera surveillance and laboratory diagnostics by 5%, underscoring the influence of ‘experience’ and potential benefits of mentorship of junior staff by senior colleagues on enhancing the implementation of these cholera interventions. The mentorship and engagement concepts also relate to the findings on previously being trained in cholera case management and participation in a cholera outbreak. Notably, both variables positively influenced HCWs’ knowledge of case management, surveillance/laboratory methods, and vaccination, and highlighting the need for continuous investment of resources for HCWs’ training and retraining in these cholera interventions.

Community-based HCWs (CHWs) were inadequate in terms of knowledge of the multi-stranded cholera interventions as compared to those occupying higher positions. This is not surprising as they are predominantly based in primary healthcare facilities with minimal or no capacity to manage cholera patients. However, community-based HCWs were significantly more knowledgeable of WASH than those occupying senior cadres, implying their strategic position in promoting infection prevention and control and strengthening healthcare system resilience in resource-constrained settings [32]. This could be because they naturally play an active role in WASH-related activities and enjoy an appreciable level of community trust and are positioned to effectively deliver risk communication in a culturally appropriate manner, thus facilitating increased access to healthcare services at the community level [43]. For example, CHEWs were integral to promoting risk communication and distributing essential supplies during a cholera outbreak in Kenya from 2014 to 2016 [44]. These attributes profoundly impact cholera control in West Africa, where sociocultural issues are highly valued [45].

This study has provided valuable evidence on HCWs’ knowledge of multi-stranded cholera interventions in cholera endemic and conflict-affected region of Nigeria. Using a policy-relevant threshold of 75 or higher score to define adequate knowledge of cholera interventions seems less prone to bias or more stable than a statistical approach (e.g., using mean value as the cut-off). The findings have also provided independent and context-specific ‘indications’ to potentially measure the impact of previous investments on HCWs' capacity strengthening in the study setting. The diversity of the study participants is also a strength of this study as it enabled us to explore the association between various professional attributes and HCWs’ knowledge of cholera interventions, which would be critical for designing and implementing cholera-focused interventions including training and retraining of HCWs.

Nonetheless, this study has some limitations worth mentioning. Firstly, without robust information on the sampling frame, HCWs’ knowledge of cholera interventions in terms of different professional cadres should be done cautiously, as those with less training, experience and occupying a position that does not require direct management of cholera cases are more likely to score lower knowledge score. Secondly, information bias is likely if there was a systematic difference in the administration of the data collection tool in Adamawa and Bauchi States, or across healthcare facilities within each state. Thirdly, by generating composite variables on knowledge of each cholera intervention, we may have missed some individual information or questions of interest; however, this approach is ideal for organising multiple variables into more digestible or meaningful information which is appealing to policymakers and health administrators [46]. Lastly, our sampling approach to selecting healthcare facilities and HCWs is prone to selection bias if the unselected facilities and HCWs were systematically different from those selected for the study.

## Conclusion

Overall, HCWs in Adamawa and Bauchi States recorded considerable knowledge of cholera interventions. While HCWs’ demographic characteristics were less important in determining their knowledge of cholera interventions, their geographic location (setting and state) and various sources of experiences (position, training and involvement in cholera outbreak response) played a predominant role.

## Supplementary Information

**Additional file 1.** Study questionnaire.

**Additional file 2.** STROBE Statement—checklist of items that should be included in reports of observational studies.

**Additional file 3.** Association between healthcare workers’ characteristics and knowledge scores for cholera multisectoral interventions.

**Additional file 4.** The unadjusted ORs for the factors associated with HCWs’ knowledge of cholera interventions.

## Data Availability

The datasets used and/or analysed during the current study are available from the corresponding author, Dr. Kelly Elimian, at Kelly.elimian@ki.se, on reasonable request.

## Abbreviations

WASH: Water, sanitation, and hygiene
OCV: Oral cholera vaccine
IDSR: Integrated Disease Surveillance and Response
WHO: World Health Organization
GTFCC: Global Task Force on Cholera Control
HCW: Healthcare worker
LGA: Local Government Area
IDPs: Internally displaced persons
IPC: Infection prevention and control
LRT: Likelihood ratio test
CHEW: Community Health Extension Worker
OR: Odds ratio
CI: Confidence intervals
