# Supplementary material for: Healthcare workers knowledge of cholera multi-stranded interventions and its determining factors in North-East Nigeria: planning and policy implications
Source: Hum Resour Health. 2023 Feb 1;21:6. doi: 10.1186/s12960-023-00796-7 (PMC9891191; doi:10.1186/s12960-023-00796-7)
Supplement: Supplementary file 1 — Additional file 1. Study questionnaire. [file 12960_2023_796_MOESM1_ESM.docx]

# Supplementary File 1

Knowledge of case management (14 questions) was assessed by asking HCWs questions around: causative microorganism of cholera; degree of cholera severity; signs and symptoms of cholera, dehydration, and severe dehydration; consequences of dehydration; clinical significance of administering oral rehydration solution; and processes and specific commodities for case management. Knowledge of WASH (7 questions) was assessed by asking questions around: benefits of adequate WASH services; recommended quantity of water per cholera patient; storage of drinking water; and infection prevention and control practices. Knowledge of surveillance (9 questions) was assessed by asking questions around: cholera case definition; functions of a surveillance system; processes and tools (forms) for surveillance; specific functions of the laboratory for cholera diagnosis; specimen for cholera diagnosis; and media for stool processing and transportation. Knowledge of coordination (2 questions) and oral cholera vaccine (5 questions) was assessed by asking questions around: activation of emergency operation centre (EOC); composition of the EOC; types of cholera vaccines; cholera vaccine dosage and interval; and preferred route of cholera vaccine administration.

| **Question** | **Possible responses** |
| --- | --- |
| **Sociodemographic characteristics** | |
| Participant ID: Date: |  |
| State: LGA: | - Adamawa - Bauchi |
| Health facility type: | - Primary - Secondary - Tertiary |
| Health facility ownership type: | - Public - Private (for profit) - Private (not for profit/NGO) |
| Setting: | - Rural - Peri-urban - Urban |
| Age in years: |  |
| Sex: | Male or female |
| Highest level of education completed | - Some primary - Primary - Some secondary - Completed secondary - Tertiary/post-secondary - Other: specify - Refused |
| Religion | - Christian - Muslim - No religion - Other: specify |
| Position: |  |
| Number of years in current role/position: |  |
| Total years of experience: |  |
| Previous training on cholera   - Yes - No - Can’t remember |  |
| If yes, specify training domain (e.g., case management/IPC, WaSH, surveillance, combined) |  |
| How many of your most recent patients (in the last 12 months) had illness that met the definition for suspected cholera? | Using since the inception of Covid-19 as a reference point |
| Any previous experience with cholera outbreak?   - No - Yes - Can’t remember |  |
| If yes, how many cholera cases on average did you manage (directly or indirectly) in a year? |  |
| **Knowledge of cholera and its interventions** | |
| ***Case management*** | |
| Cholera infection is often   - Severe - Mild - Don’t know | *Mild* |
| About 1 in 10 people with cholera will experience severe symptoms, which, in the early stages, include:   - Profuse watery diarrhoea (sometimes described as rice-water stools) - Vomiting - Thirst - Leg cramps - Restlessness or irritability - Cough - Do not know | *Any specific option but cough and don’t know* |
| Health care workers are expected to look out for signs of dehydration when examining a suspected cholera patient. These signs include:   - Restless, irritable - Eyes are sunken - Absence of tears in the eyes - Mouth and tongue are dry - Thirsty, drink water eagerly - Skin pinch goes back slowly - Fever - Do not know | *Any specific option but fever and don’t know* |
| Signs of severe dehydration (*multiple answers acceptable*) include the following except?   - Lethargic or unconscious, floppy - Eyes are very sunken & dry - Bleeding or loss of blood - Absence of tears in the eyes - Mouth and tongue are very dry - Drink poorly or unable to drink - Skin pinch goes back very slowly - Do not know | *Any specific option but bleeding or loss of blood and don’t know* |
| What are the possible consequences of dehydration in the course of cholera infection?   - Unconsciousness due to severe dehydration - Kidney failure - Heart attack or cardiac arrest - Death - Do not know | *Any specific option but heart attack and don’t know* |
| If left untreated, severe dehydration can lead to shock, coma, and death within hours.   - False - True | *True* |
| Most patients with mild cholera infection can be treated with both oral rehydration solution and administration of intravenous fluid.   - True - False | *False*  *Most patients can be treated using oral rehydration solution alone. Only severely dehydrated patients need the administration of intravenous fluid.* |
| The most important element of cholera treatment is rapid replacement of the water and salts lost through diarrhoea and vomiting.   - True - False | *True* |
| Patients with severe dehydration and/or uncontrollable vomiting can be managed at home with oral rehydration solution.   - True - False | *False. They must be admitted to a hospital for treatment with ORS* |
| The process of reserving relevant stock of supplies needed for prompt response to cholera outbreaks is called?   - Storage - Pre-positioning | *Pre-positioning* |
| The selection of antimicrobials for cholera treatment should be based on recent susceptibility testing.   - False - True | *True* |
| People with cholera infection at risk of severe illness or death include all but the following:   - Those with diarrhoea who do not seek treatment until they are severely dehydrated - A cholera patient whose complications are recognized early - Cholera patients who take the wrong antibiotics (whether from a doctor, a pharmacist or a drug vendor) | *All but the second/middle option* |
| Cholera is caused by the ingestion of food or water contaminated with *V. cholerae*   - False - True | *True* |
| What type of treatment should be given to a cholera patient? (Multiple answers acceptable)   - Packet saline - Rice saline - IV saline - Home-made saline - Zink tablet or syrup - Antibiotics - Herbs - Plain water - Nothing or watch and pray - Do not know | *Packet saline*  *Rice saline*  *IV saline*  *Home-made saline*  *Zink tablet or syrup*  *Antibiotics*  *Plain water* |
| ***WASH*** | |
| The following persons are at risk of contracting cholera except:   - Those who do not wash their hands when and as they should (before eating, after defecation or after contact with stools) - Those who do not dispose of stools properly - Those who do not consume safe water or store it properly - Those who cook or eat food safely | *All but the last option* |
| Effective cholera control measures include the following excerpt:   - Ensuring a safe supply of water - Food safety - Hand washing - Environmental sanitation - Safe practices at funerals - Travel and international trade restrictions | *All but the last option* |
| There should be 40-60 litres of water available per patient per day for drinking, cleaning, bathing, and washing clothes, to prevent cholera transmission.   - True - False | *True* |
| Drinking-water should not be stored separately from water for other uses.   - False - True | *False* |
| Soiled bedding, clothing and other articles are washed and disinfected frequently in any area of a health facility.   - True - False | *False, this should be done only in a designated area* |
| What are the common cholera prevention measures? (multiple answers acceptable)   - Use of safe water for drinking & household works - Provision of safe drinking water supply - Use of sanitary latrine - Satisfactory sewage system/ proper sanitary disposal of excreta, refuse - Washing hands with soap before meal or after defecation - Early case finding in the community - Disinfection of all the contaminated articles with clean water - Good hygiene practice - Health education - Oral cholera vaccine - Do not know | Any specific option but disinfection of all contaminated articles with water—water is not good for disinfection purposes. |
| Each patient lies on a pierced bed with a bucket for both stool and vomit collection underneath   - True - False | *False*  *Each patient lies on a pierced bed with a bucket for stool collection underneath + a separate bucket for vomit besides the bed* |
| ***Surveillance (epidemiology and laboratory)*** | |
| Cholera case definition: Suspect a cholera infection if:   - A patient older than 5 years develops severe dehydration or dies of acute watery diarrhoea - There is a sudden increase in the daily number of patients with acute watery diarrhoea, especially if there is evidence of “rice water stools” - Presentation with cough, fever and diarrhoea | Any of the first two definitions is correct |
| Surveillance allows health workers to (Tick all that are applicable):   - Detect outbreaks early - Generate funding - Estimate how many people become sick and die - Know when and where a disease occurs - Determine if a disease is spreading - Estimate supplies and staff needed for a disease outbreak - Evaluate whether control measures are successful | *All but the second options are applicable* |
| When a person with suspected cholera is seen at a health facility, information about the person may be recorded in:   - Patient register - A tally form - A weekly / monthly report form - Notifiable/epidemic diseases form | *Patient register and/or notifiable/epidemic disease form* |
| The process of recording a suspected cholera patient’s detail on the surveillance system is called?   - Recording - Line listing | *Line listing* |
| The role of a health worker in cholera surveillance is to:   - Fill out and send in forms promptly - Interpret information and act on it - Try to understand the disease | *All but the last option* |
| A common medium for the transportation of stool specimen to the lab for diagnosis is called:  *Allow the health worker to specify the name* | *Cary Blair Medium* |
| If Cary-Blair transport medium is not available, alkaline peptone water can be used to transport the specimen, as long as the specimen is likely to reach the laboratory and be processed within 24 hours.   - True - False | True |
| For cholera, the role of the laboratory is to:   - Be actively involved in risk communication - Isolate the organism when an epidemic is first reported - Determine the antibiotic sensitivity of the organism - Monitor the antibiotic sensitivity regularly during the epidemic | *All but the first option* |
| What specimen is used for cholera diagnosis?   - Blood test - Stool sample - Nasopharyngeal and oropharyngeal samples - Don’t know | *Stool sample is used for lab diagnosis* |
| ***Coordination*** | |
| The emergency Operation Centre is usually activated during routine cholera surveillance   - False - True | *False*  *The EOC is activated during a disease outbreak* |
| Which response pillar/unit typically form an EOC? (multiple responses allowed)   - Presidential task force - Surveillance/laboratory - Case management - IPC - Coordination - Risk communication/community engagement - Logistics | *Any specific option but the presidential task force* |
| ***Cholera vaccination*** | |
| Which of the following is/are a cholera vaccine?   - Shanchol - Dukoral - Euvichol-Plus/Euvichol - Vaxchora - Cholechol | *All but Cholechol (one correct option is considered correct)* |
| What is the required number of doses of cholera vaccine?  ………………(#)  Do not know | *All but Vaxchora require 2 doses for full protection* |
| What is the minimum interval between doses of cholera vaccine?  ................... (weeks)  Do not know | *Typically 1-2 weeks delay between each dose* |
| What is the preferred route for the administration of cholera vaccine?   - Oral - Through injection - Don’t know | *Oral* |
| Cholera vaccines offer complete protection, with effectiveness increasing with time   - True, protection is permanent - False, protection is time-dependent | *True* |
