# Supplementary material for: Healthcare workers knowledge of cholera multi-stranded interventions and its determining factors in North-East Nigeria: planning and policy implications
Source: Hum Resour Health. 2023 Feb 1;21:6. doi: 10.1186/s12960-023-00796-7 (PMC9891191; doi:10.1186/s12960-023-00796-7)
Supplement: Supplementary file 3 — Additional file 3. Association between healthcare workers’ characteristics and knowledge scores for cholera multisectoral interventions. [file 12960_2023_796_MOESM3_ESM.docx]

**Supplementary File 3**

**Association between healthcare workers’ characteristics and knowledge scores for cholera multisectoral interventions**

|  | | | | |
| --- | --- | --- | --- | --- |
| **Variable** | **Case management** | | | |
|  | **Good score**  **[n=257]**  **(n, %)** | **Poor score**  **[n=233]**  **(n, %)** | **Overall score**  **[N=490]**  **(N, %)** | **P-value** |
| **State**  Adamawa  Bauchi | 114 (44.36)  143 (55.64) | 140 (60.09)  93 (39.91) | 254 (51.84)  236 (48.16) | 0.001 |
| **Age (SD), year** | 35.51 (7.89) | 35.42 (8.06) | 35.46 (7.97) |  |
| **Sex**  Female  Male | 135 (52.53)  122 (47.47) | 93 (39.91)  140 (60.09) | 228 (46.53)  262 (53.47) | 0.005 |
| **Health facility type**  Primary  Secondary  Tertiary | 215 (83.66)  38 (14.79)  4 (1.56) | 201 (86.27)  26 (11.16)  6 (2.58) | 416 (84.90)  64 (13.06)  10 (2.04) | 0.377 |
| **Setting**  Rural  Urban  Peri-urban | 130 (50.58)  110 (42.80)  17 (6.61) | 87 (37.34)  115 (49.36)  31 (13.30) | 217 (44.29)  225 (45.92)  48 (9.80) | 0.003 |
| **Highest level of education completed**  Some primary/primary  Some secondary/secondary  Post-secondary/tertiary  Other (e.g., Almajiranci) | 60 (23.35)  17 (6.61)  177 (68.87)  3 (1.17) | 17 (7.30)  33 (14.16)  181 (77.68)  2 (0.86) | 77 (15.71)  50 (10.20)  358 (73.06)  5 (1.02) | <0.001 |
| **Religion**  Christian  Muslim  Non-religious | 125 (48.64)  129 (50.19)  3 (1.17) | 90 (38.63)  142 (60.94)  1 (0.43) | 215 (43.88)  271 (55.31)  4 (0.82) | 0.046 |
| **Current position of HCW**  CHEW/CHO/attendant/cleaner/casual staff  Junior: disease focal person/dispenser  Nurse/laboratorian/data scientist  Senior: Clinician/administrative/chief nurse | 81 (31.52)  74 (28.79)  27 (10.51)  71 (27.63) | 108 (45.92)  27 (11.59)  47 (20.17)  44 (18.88) | 188 (38.37)  101 (20.61)  74 (15.10)  115 (23.47) | <0.001 |
| **Duration in current position, year** | 12.54 (12.22) | 7.19 (7.63) | 10.00 (10.63) |  |
| **Previous training on cholera management**  No  Yes | 172 (66.93)  85 (33.07) | 200 (85.84)  33 (14.16) | 372 (75.92)  118 (24.08) | <0.001 |
| **Previous response to a cholera outbreak**  No  Yes | 86 (33.46)  171 (66.54) | 129 (55.36)  104 (44.64) | 215 (43.88)  275 (56.12) | <0.001 |

|  | | | | |
| --- | --- | --- | --- | --- |
| **Variable** | **WASH** | | | |
|  | **Good score**  **[n=344]**  **(n, %)** | **Poor score**  **[n=146]**  **(n, %)** | **Overall score**  **[N=490]**  **(N, %)** | **P-value** |
| **State**  Adamawa  Bauchi | 168 (48.84)  176 (51.16) | 86 (58.90)  60 (41.10) | 254 (51.84)  236 (48.16) | 0.041 |
| **Age, year** | 35.94 (7.99) | 34.34 (7.83) | 35.46 (7.97) |  |
| **Sex**  Female  Male | 166 (48.26)  178 (51.74) | 62 (42.47)  84 (57.53) | 228 (46.53)  262 (53.47) | 0.240 |
| **Health facility type**  Primary  Secondary  Tertiary | 291 (84.59)  47 (13.66)  6 (1.74) | 125 (85.62)  17 (11.64)  4 (2.74) | 416 (84.90)  64 (13.06)  10 (2.04) | 0.660 |
| **Setting**  Rural  Urban  Peri-urban | 153 (44.48)  156 (45.35)  35(10.17) | 64 (43.84)  69 (47.26)  13 (8.90) | 217 (44.29)  225 (45.92)  48(9.80) | 0.878 |
| **Highest level of education completed**  Some primary/primary  Some secondary/secondary  Post-secondary/tertiary  Other (e.g., Almajiranci) | 62 (18.02)  23 (6.69)  255 (74.13)  4 (1.16) | 15 (10.27)  27 (18.49)  103 (70.55)  1 (0.68) | 77 (15.71)  50 (10.20)  358 (73.06)  5 (1.02) | <0.001 |
| **Current position of HCW**  CHEW/CHO/attendant/cleaner/casual staff  Junior: disease focal person/dispenser  Nurse/laboratorian/data scientist  Senior: Clinician/administrative/chief nurse | 129 (37.50)  70 (20.35)  51 (14.83)  88 (25.58) | 59 (40.41)  31 (21.23)  23 (15.75)  27 (18.49) | 188 (38.37)  101 (20.61)  74 (15.10)  115 (23.47) | 0.302 |
| **Duration in current position, year** | 11.26 (11.37) | 7.03 (7.90) | 10.00 (10.63) |  |
| **Previous training on cholera management**  No  Yes | 245 (71.22)  99 (28.78) | 127 (86.99)  19 (13.01) | 372 (75.92)  118 (24.08) | <0.001 |
| **Previous response to a cholera outbreak**  No  Yes | 131 (38.08)  213 (61.92) | 84 (57.53)  62 (42.47) | 215 (43.88)  275 (56.12) | <0.001 |

|  | | | | |
| --- | --- | --- | --- | --- |
| **Variable** | **Surveillance and laboratory** | | | |
|  | **Good score**  **[n=258]**  **(n, %)** | **Poor score**  **[n=232]**  **(n, %)** | **Overall score**  **[N=490]**  **(N, %)** | **P-value** |
| **State**  Adamawa  Bauchi | 134 (51.94)  124 (48.06) | 120 (51.72)  112 (48.28) | 254 (51.84)  236 (48.16) | 0.962 |
| **Age, year** | 36.24 (8.53) | 34.60 (7.20) | 35.46 (7.97) |  |
| **Sex**  Female  Male | 126 (48.84)  132 (51.16) | 102 (43.97)  130 (56.03) | 228 (46.53)  262 (53.47) | 0.280 |
| **Health facility type**  Primary  Secondary  Tertiary | 206 (79.84)  46 (17.83)  6 (2.33) | 210 (90.52)  18 (7.76)  4 (1.72) | 416 (84.90)  64 (13.06)  10 (2.04) | 0.003 |
| **Setting**  Rural  Urban  Peri-urban | 99 (38.37)  134 (51.94)  25 (9.69) | 118 (50.86)  91 (39.22)  23 (9.91) | 217 (44.29)  225 (45.92)  48 (9.80) | 0.014 |
| **Highest level of education completed**  Some primary/primary  Some secondary/secondary  Post-secondary/tertiary  Other (e.g., Almajiranci) | 8 (3.10)  23 (8.91)  225 (87.21)  2 (0.78) | 69 (29.74)  27 (11.64)  133 (57.33)  3 (1.29) | 77 (15.71)  50 (10.20)  358 (73.06)  5 (1.02) | <0.001 |
| **Current position of HCW**  CHEW/CHO/attendant/cleaner/casual staff  Junior: disease focal person/dispenser  Nurse/laboratorian/data scientist  Senior: Clinician/administrative/chief nurse | 40 (15.50)  79 (30.62)  45 (17.44)  89 (34.50) | 148 (63.79)  22 (9.48)  29 (12.50)  26 (11.21) | 188 (38.37)  101 (20.61)  74 (15.10)  115 (23.47) | <0.001 |
| **Duration in current position, year** | 7.86 (7.92) | 12.38 (12.59) | 10.00 (10.63) |  |
| **Previous training on cholera management**  No  Yes | 167 (64.73)  91 (35.27) | 205 (88.36)  27 (11.64) | 372 (75.92)  118 (24.08) | <0.001 |
| **Previous response to a cholera outbreak**  No  Yes | 73 (28.29)  185 (71.71) | 142 (61.21)  90 (38.79) | 215 (43.88)  275 (56.12) | <0.001 |

|  | | | | |
| --- | --- | --- | --- | --- |
| **Variable** | **Coordination mechanism** | | | |
|  | **Good score**  **[n=271]**  **(n, %)** | **Poor score**  **[n=219]**  **(n, %)** | **Overall score**  **[N=490]**  **(N, %)** | **P-value** |
| **State**  Adamawa  Bauchi | 155 (57.20)  116 (42.80) | 99 (45.21)  120 (54.79) | 254 (51.84)  236 (48.16) | 0.008 |
| **Age, year** | 35.29 (8.17) | 35.67 (7.71) | 35.46 (7.97) |  |
| **Sex**  Female  Male | 136 (50.18)  135 (49.82) | 92 (42.01)  127 (57.99) | 228 (46.53)  262 (53.47) | 0.071 |
| **Health facility type**  Primary  Secondary  Tertiary | 216 (79.70)  45 (16.61)  10 (3.69) | 200 (91.32)  19 (8.68)  0 (0.00) | 416 (84.90)  64 (13.06)  10 (2.04) | <0.001 |
| **Setting**  Rural  Urban  Peri-urban | 100 (36.90)  144 (53.14)  27 (9.96) | 117 (53.42)  81 (36.99)  21 (9.59) | 217 (44.29)  225 (45.92)  48 (9.80) | 0.001 |
| **Highest level of education completed**  Some primary/primary  Some secondary/secondary  Post-secondary/tertiary  Other (e.g., Almajiranci) | 44 (16.24)  21 (7.75)  204 (75.28)  2 (0.74) | 33 (15.07)  29 (13.24)  154 (70.32)  3 (1.37) | 77 (15.71)  50 (10.20)  358 (73.06)  5 (1.02) | 0.206 |
| **Current position of HCW**  CHEW/CHO/attendant/cleaner/casual staff  Junior: disease focal person/dispenser  Nurse/laboratorian/data scientist  Senior: Clinician/administrative/chief nurse | 82 (30.26)  79 (29.15)  39 (14.39)  66 (24.35) | 106 (48.40)  22 (10.05)  35 (15.98)  49 (22.37) | 188 (38.37)  101 (20.61)  74 (15.10)  115 (23.47) | <0.001 |
| **Duration in current position, year** | 10.34 (11.46) | 9.58 (9.50) | 10.00 (10.63) |  |
| **Previous training on cholera management**  No  Yes | 180 (66.42)  91 (33.58) | 192 (87.67)  27 (12.33) | 372 (75.92)  118 (24.08) | <0.001 |
| **Previous response to a cholera outbreak**  No  Yes | 94 (34.69)  177 (65.31) | 121 (55.25)  98 (44.75) | 215 (43.88)  275 (56.12) | <0.001 |

|  | | | | |
| --- | --- | --- | --- | --- |
| **Variable** | **Oral cholera vaccination** | | | |
|  | **Good score**  **[n=239]**  **(n, %)** | **Poor score**  **[n=251]**  **(n, %)** | **Overall score**  **[N=490]**  **(N, %)** | **P-value** |
| **State**  Adamawa  Bauchi | 71 (29.71)  168 (70.29) | 183 (72.91)  68 (27.09) | 254 (51.84)  236 (48.16) | <0.001 |
| **Age, year** | 36.61 (7.25) | 34.37 (8.46) | 35.46 (7.97) |  |
| **Sex**  Female  Male | 100 (41.84)  139 (58.16) | 128 (51.00)  123 (49.00) | 228 (46.53)  262 (53.47) | 0.042 |
| **Health facility type**  Primary  Secondary  Tertiary | 208 (87.03)  28 (11.72)  3 (1.26) | 208 (82.87)  36 (14.34)  7 (2.79) | 416 (84.90)  64 (13.06)  10 (2.04) | 0.315 |
| **Setting**  Rural  Urban  Peri-urban | 131 (54.81)  85 (35.56)  23 (9.62) | 86 (34.26)  140 (55.78)  25 (9.96) | 217 (44.29)  225 (45.92)  48 (9.80) | <0.001 |
| **Highest level of education completed**  Some primary/primary  Some secondary/secondary  Post-secondary/tertiary  Other (e.g., Almajiranci) | 52 (21.76)  13 (5.44)  170 (71.13)  4 (1.67) | 25 (9.96)  37 (14.74)  188 (74.90)  1 (0.40) | 77 (15.71)  50 (10.20)  358 (73.06)  5 (1.02) | <0.001 |
| **Current position of HCW**  CHEW/CHO/attendant/cleaner/casual staff  Junior: disease focal person/dispenser  Nurse/laboratorian/data scientist  Senior: Clinician/administrative/chief nurse | 102 (42.68)  35 (14.64)  26 (10.88)  71 (29.71) | 86 (34.26)  66 (26.29)  48 (19.12)  44 (17.53) | 188 (38.37)  101 (20.61)  474 (15.10)  115 (23.47) | <0.001 |
| **Duration in current position, year** | 13.71 (11.72) | 6.47 (8.03) | 10.00 (10.63) |  |
| **Previous training on cholera management**  No  Yes | 167 (69.87)  72 (30.13) | 205 (81.67)  46 (18.33) | 372 (75.92)  118 (24.08) | 0.002 |
| **Number of cholera patients managed in the last 12 months**  None  1-3 patients  ≥4 patients | 209 (87.45)  16 (6.69)  14 (5.86) | 230 (91.63)  15 (5.98)  6 (2.39) | 439 (89.59)  31 (6.33)  20 (4.08) | 0.139 |
| **Previous response to a cholera outbreak**  No  Yes | 104 (43.51)  135 (56.49) | 111 (44.22)  140 (55.78) | 215 (43.88)  275 (56.12) | 0.874 |
