# Supplementary material for: Healthcare workers knowledge of cholera multi-stranded interventions and its determining factors in North-East Nigeria: planning and policy implications
Source: Hum Resour Health. 2023 Feb 1;21:6. doi: 10.1186/s12960-023-00796-7 (PMC9891191; doi:10.1186/s12960-023-00796-7)
Supplement: Supplementary file 4 — Additional file 4. The unadjusted ORs for the factors associated with HCWs’ knowledge of cholera interventions. [file 12960_2023_796_MOESM4_ESM.docx]

**Supplementary File 4**

**The unadjusted ORs for the factors associated with HCWs’ knowledge of cholera interventions**

*Case management*

HCWs’ knowledge of case management was significantly associated with sex, setting, current position, previous training in cholera case management, and previous participation in a cholera outbreak. Specifically, being male and residing in urban and peri-urban areas were associated with increased odds of low knowledge of cholera case management. In contrast, knowledge of cholera case management significantly increased with being a junior (disease focal person/dispenser) staff and a senior (administrative/chief nurse) staff as well as previously receiving training in cholera case management and previously participating in a cholera outbreak.

*WASH*

Unlike case management, fewer variables showed significant association with HCWs’ knowledge of WASH. Being a junior (disease focal person/dispenser) staff, a nurse/laboratorian/data scientist, and a senior (administrative/chief nurse) staff was associated with higher odds of inadequate knowledge of WASH than being a CHEW/CHO/attendant/cleaner/casual staff. However, a 1-year stay in the current position significantly decreased the odds of inadequate knowledge of WASH by 3%.

*Surveillance and laboratory*

Age, health facility type, setting, educational attainment, current position, duration in current position, previous training in cholera management, and previous participation in a cholera outbreak were all significantly associated with HCWs’ knowledge of cholera surveillance and laboratory diagnosis. Apart from a 1-year stay in the current position which appeared to increase the odds of inadequate knowledge of surveillance and laboratory by 3%, the remaining favourable appear to have favoured adequate knowledge of cholera surveillance and laboratory.

*Coordination*

HCWs’ gender, health facility type, setting, educational attainment, current position, duration in current position, previous training in cholera management, and previous response to a cholera outbreak were all significantly associated with their knowledge of cholera outbreak coordination. Other than male gender and duration in current position which increased the odds of inadequate knowledge of cholera outbreak coordination, the other variables had a favourable impact on the outcome variable.

*Oral cholera vaccine*

Other than residency in urban area which increased the odds of inadequate knowledge of OCV by 61%, residency in Bauchi State, increasing age, post-secondary/tertiary education, junior and senior staff, previous training in cholera case management, and previous participation in a cholera outbreak significantly lowered the odds of inadequate knowledge of OCV.

| **Table 4: Factors associated with inadequate knowledge of cholera interventions** | | | | | |
| --- | --- | --- | --- | --- | --- |
| **Variable** | **Case management** | **WASH** | **Surveillance & laboratory** | **Coordination** | **Oral cholera vaccine** |
|  | **Unadjusted OR (95% CI)** | | | | |
| **State**  Adamawa  Bauchi | 1.00  0.97 (0.64-1.45) NS | 1.00  0.78 (0.45-1.37) NS | 1.00  0.89 (0.62-1.27) NS | 1.00  1.37 (0.93-2.04) NS | 1.00  **0.18 (0.10-0.31)** ‡ |
| **Age, year** | 1.00 (0.98-1.03) NS | 1.01 (0.97-1.04) NS | **0.97 (0.95-0.99)** † | 0.99 (0.97-1.02) NS | **0.95 (0.93-0.98)** † |
| **Sex**  Female  Male | 1.00  **1.59 (1.06-2.39)** † | 1.00  1.17 (0.67-2.04) NS | 1.00  1.11 (0.78-1.58) NS | 1.00  **1.58 (1.06-2.34)** † | 1.00  0.73 (0.46-1.16) NS |
| **Health facility type**  Primary  Secondary  Tertiary | 1.00  0.75 (0.42-1.33)  3.08 (0.39-24.58) NS | 1.00  1.09 (0.47-2.52)  Omitted NS | 1.00  **0.47 (0.27-0.80)**  0.73 (0.21-2.55) † | 1.00  **0.36 (0.21-0.62)**  0.51 (0.14-1.85) † | 1.00  0.73 (0.39-1.37)  2.01 (0.25-16.12) NS |
| **Setting**  Rural  Urban  Peri-urban | 1.00  **1.05 (0.69-1.60)**  **3.36 (1.27-8.89)** † | 1.00  0.73 (0.41-1.32)  1.24 (0.41-3.78) NS | 1.00  **0.64 (0.44-0.93)**  1.00 (0.53-1.90) † | 1.00  **0.46 (0.30-0.71)**  **0.46 (0.24-0.91)** † | 1.00  **1.61 (1.00-2.59)**  2.64 (0.99-7.02) † |
| **Highest level of education**  Primary  Secondary  Post-secondary/tertiary  Other | 1.00  0.67 (0.29-1.54)  0.72 (0.39-1.30)  1.05 (0.11-10.05) NS | 1.00  2.56 (0.79-8.27)  1.93 (0.98-3.77)  0.89 (0.09-8.57) NS | 1.00  **0.10 (0.03-0.28)**  **0.06 (0.02-0.15)**  0.10 (0.01-1.77) ‡ | 1.00  **0.20 (0.07-0.55)**  **0.17 (0.07-0.40)**  0.34 (0.03-3.52) † | 1.00  1.32 (0.23-7.46)  **0.18 (0.06-0.50)**  **0.08 (0.01-0.64)** † |
| **Current position (cadre)ֆ**  1  2  3  4  5 | 1.00  **0.09 (0.05-0.17)**  0.72 (0.32-1.63)  Omitted  **0.20 (0.11-0.36)** ‡ | 1.00  **4.55 (1.72-11.99)**  **2.68 (1.08-6.67)**  Omitted  **2.79 (1.29-6.03)** ‡ | 1.00  **0.06 (0.03-0.11)**  **0.22 (0.12-0.40)**  0.35 (0.10-1.24)  **0.08 (0.05-0.14)** ‡ | 1.00  **0.07 (0.04-0.13)**  0.51 (0.24-1.07)  0.60 (0.12-2.91)  **0.21 (0.12-0.39)** † | 1.00  **0.49 (0.24-0.97)**  0.81 (0.35-1.89)  1.24 (0.15-10.11)  **0.18 (0.10-0.33)** † |
| **Duration in current position, year** | 0.99 (0.98-1.01) NS | **0.97 (0.94-0.99)** † | **1.03 (1.01-1.05)** ‡ | **1.03 (1.01-1.05)** † | 0.98 (0.96-1.00) NS |
| **Previous training in cholera management**  No  Yes | 1.00  **0.29 (0.19-0.45)** ‡ | 1.00  1.18 (0.60-2.33) NS | 1.00  **0.24 (0.16-0.38)** ‡ | 1.00  **0.44 (0.29-0.68)** ‡ | 1.00  **0.25 (0.15-0.40)** † |
| **Previous participation in a cholera outbreak**  No  Yes | 1.00  **0.24 (0.15-0.38)** ‡ | 1.00  0.95 (0.54-1.67) NS | 1.00  **0.21 (0.14-0.32)** ‡ | 1.00  **0.31 (0.20-0.48)** ‡ | 1.00  **0.27 (0.16-0.46)** ‡ |
| †=P-value <0.05; ‡=P-value <0.001; NS=P-value not statistically significant (i.e., ≥0.05)  ֆCurrent position: 1=CHEW/CHO/attendant/cleaner/casual staff; 2= junior (disease focal person/dispenser) staff; 3= nurse/laboratorian/data scientist; 4=clinician/pharmacist; 5=senior (administrative/chief nurse) staff  Wald’s p-values are presented for binary and continuous variables; LRT p-values are presented for categorical variables  Statistically significant results are in **bold**. | | | | | |
